# A receptor-based analysis of local ecosystems in the human brain

**DOI:** 10.1186/s12868-017-0355-2

**Published:** 2017-03-20

**Authors:** Skirmantas Janušonis

**Affiliations:** Department of Psychological and Brain Sciences, University of California, Santa Barbara, CA 93106-9660 USA

**Keywords:** Neural networks, Ecosystems, Neurotransmitter receptors, Forebrain, Midbrain, Hindbrain, Pallium, Cerebral cortex, Hierarchical clustering, Multiscale bootstrap resampling

## Abstract

**Background:**

As a complex system, the brain is a self-organizing entity that depends on local interactions among cells. Its regions (anatomically defined nuclei and areas) can be conceptualized as cellular ecosystems, but the similarity of their functional profiles is poorly understood. The study used the Allen Human Brain Atlas to classify 169 brain regions into hierarchically-organized environments based on their expression of 100 G protein-coupled neurotransmitter receptors, with no a priori reference to the regions’ positions in the brain’s anatomy or function. The analysis was based on hierarchical clustering, and multiscale bootstrap resampling was used to estimate the reliability of detected clusters.

**Results:**

The study presents the first unbiased, hierarchical tree of functional environments in the human brain. The similarity of brain regions was strongly influenced by their anatomical proximity, even when they belonged to different functional systems. Generally, spatial vicinity trumped long-range projections or network connectivity. The main cluster of brain regions excluded the dentate gyrus of the hippocampus. The nuclei of the amygdala formed a cluster irrespective of their striatal or pallial origin. In its receptor profile, the hypothalamus was more closely associated with the midbrain than with the thalamus. The cerebellar cortical areas formed a tight and exclusive cluster. Most of the neocortical areas (with the exception of some occipital areas) clustered in a large, statistically well supported group that included no other brain regions.

**Conclusions:**

This study adds a new dimension to the established classifications of brain divisions. In a single framework, they are reconsidered at multiple scales—from individual nuclei and areas to their groups to the entire brain. The analysis provides support for predictive models of brain self-organization and adaptation.

## Background

The brain can be perceived as a well-tuned machine. In this view, each of its parts performs specific, pre-assigned computations (subroutines), which then are linked through local and long-range neuroanatomical connections. Throughout the history of neuroscience, this intuition has been supported by human-made devices that are composed of functionally-dedicated, irreplaceable, and permanently connected parts. The value of this approach has been demonstrated by experimental and clinical observations in specific brain subsystems (particularly sensory and motor), and it remains a powerful guiding principle in basic research and clinical practice.

At a deeper conceptual level, the brain is very different from human-made machines. It is a complex and adaptive dynamical system [1] that is not fundamentally different from other living systems such as individual cells, ecosystems, or human societies. In all of these self-organizing (“dissipative”) systems [2, 3], the global order is not the master that drives the parts but rather a product (“emergent phenomenon”) of local interactions. The simplicity of these local processes often defies intuition [4–8]. The potential of complex-systems approaches in neuroscience has been appreciated for decades [9, 10], but it is only recently that they have begun to reshape our understanding of global brain properties, such as large-scale regional networks [11–13], neurotransmitter receptor communities [14], and nested cortical oscillations [15].

It is natural to expect that self-organizing forces operate at the level of local cellular ecosystems, corresponding to anatomically defined brain regions. These regions are often assigned a specific role based on their distinguishing characteristics (e.g., the “serotonergic” raphe nuclei or the “motor” cortex). In reality, each brain region is a rich, adapting system containing diverse populations of cells and a multitude of other elements (traversing axons, the extracellular matrix, blood cells and cell fragments [16], extracellular vesicles [17]). The internal richness of these systems, the core of their self-organization, is typically poorly understood and is often obscured by their assigned “function.” Treating them as ecosystems does not merely replace one metaphor with another metaphor; instead, it leads to specific predictions. In particular, it suggests that brain cells may support one another only when the benefits of cooperation outweigh competition, axon and vasculature routing may be only locally beneficial (with no meaningful global function), some cell groups in the healthy brain may be essentially parasitic (with other cells simply adapting to them), and the entire brain may be a “tensegrity” structure composed of these opposing forces. Scattered evidence from various brain systems supports this possibility [18–21]. Importantly, different brain regions may represent the same “neurobiome,” just as two separated geographical areas may represent the same ecological biome (e.g., tropical rainforest, savanna).

Direct observation of interactions among many diverse elements in a brain region is beyond our current technical capabilities. However, high-throughput analyses already allow us to “phenotype” cellular ecosystems, with no reference to their functions (real or perceived). This analysis can be based on neurotransmitter receptors, a well understood element of brain dynamics. The purpose of neurotransmitter receptors, or any sensors in general, is to detect a change in time (an always-present entity carries no information and its detection is wasteful). The carrier of the change is represented by the general receptor class (e.g., receptors detect a concentration difference of glutamate but not dopamine). Within a receptor class, a receptor subtype (e.g., mGluR1) is likely to be associated with a particular dynamic of the change. This aspect of neurotransmitter signaling has received surprisingly little attention, even though it can be built on a solid theoretical foundation. Narrow tuning based on expected signal patterns increases detection sensitivity, boosts processing speed, and reduces energy consumption. This Bayesian property has been demonstrated in the spiking of some neurons [22], in brain sensory systems [11], and it may also operate at the level of single but complex molecules. In particular, different receptor subtypes may be sensitive to different dynamical patterns of the same physical carriers. This can explain the multitude of receptor subtypes, many of which converge onto the same signaling cascades. As a consequence, the expression levels of specific receptor subtypes may reflect the (directly unobservable) neurotransmitter dynamics in the given brain region. In addition, some receptor subtypes can be cell-type specific and represent the cellular composition of the region.

These considerations suggest that, if many receptors are considered simultaneously, their set can provide a reasonable approximation of the structure and natural dynamics of a brain region. Importantly, this approximation is unbiased with respect to the proposed “function” of the region. This study sought to hierarchically classify 169 regions of the human brain, based on their expression of a large set of G protein-coupled neurotransmitter receptors (GPCRs).

## Methods

The mRNA expression data (*z*-scores) of 100 GPCRs (Table 1) in 169 brain regions of 6 human brain specimens were downloaded from the Allen Brain Atlas data portal (http://human.brain-map.org; April 23, 2016). The brain donors were two African-American males (24 and 39 years of age), three Caucasian males (31, 55, and 57 years of age), and one Hispanic female (49 years of age). Technical details about the brain donors, tissue preparation, specificity controls, and data normalization are described in the Allen Human Brain Atlas Technical White Papers (Case Qualification and Donor Profiles, Microarray Survey, Microarray Data Normalization).

**Table 1:** The receptor set

Number	Neurotransmitter	Receptor	Gene
1	Glutamate	mGluR1	GRM1
2	Glutamate	mGluR2	GRM2
3	Glutamate	mGluR3	GRM3
4	Glutamate	mGluR4	GRM4
5	Glutamate	mGluR5	GRM5
6	Glutamate	mGluR6	GRM6
7	Glutamate	mGluR7	GRM7
8	Glutamate	mGluR8	GRM8
9	GABA	GABABR1	GABBR1
10	GABA	GABABR2	GABBR2
11	Dopamine	D1	DRD1
12	Dopamine	D2	DRD2
13	Dopamine	D3	DRD3
14	Dopamine	D4	DRD4
15	Dopamine	D5	DRD5
16	Adrenergic	α1A	ADRA1A
17	Adrenergic	α1B	ADRA1B
18	Adrenergic	α1D	ADRA1D
19	Adrenergic	α2A	ADRA2A
20	Adrenergic	α2B	ADRA2B
21	Adrenergic	α2C	ADRA2C
22	Adrenergic	β1	ADRB1
23	Adrenergic	β2	ADRB2
24	Adrenergic	β3	ADRB3
25	Serotonin	5-HT1A	HTR1A
26	Serotonin	5-HT1B	HTR1B
27	Serotonin	5-HT1D	HTR1D
28	Serotonin	5-HT1E	HTR1E
29	Serotonin	5-HT1F	HTR1F
30	Serotonin	5-HT2A	HTR2A
31	Serotonin	5-HT2B	HTR2B
32	Serotonin	5-HT2C	HTR2C
33	Serotonin	5-HT4	HTR4
34	Serotonin	5-HT5A	HTR5A
35	Serotonin	5-HT6	HTR6
36	Serotonin	5-HT7	HTR7
37	Cholinergic	M1	CHRM1
38	Cholinergic	M2	CHRM2
39	Cholinergic	M3	CHRM3
40	Cholinergic	M4	CHRM4
41	Cholinergic	M5	CHRM5
42	Histamine	H1	HRH1
43	Histamine	H2	HRH2
44	Histamine	H3	HRH3
45	Histamine	H4	HRH4
46	Bradykinin	B1	BDKRB1
47	Bradykinin	B2	BDKRB2
48	Cholecystokinin	CCK1	CCKAR
49	Cholecystokinin	CCK2	CCKBR
50	CRH	CRF1	CRHR1
51	CRH	CRF2	CRHR2
52	Galanin	Gal1	GALR1
53	Galanin	Gal2	GALR2
54	Galanin	Gal3	GALR3
55	MCH	MCH1	MCHR1
56	MCH	MCH2	MCHR2
57	MSH	MC1	MC1R
58	MSH	MC2	MC2R
59	MSH	MC3	MC3R
60	MSH	MC4	MC4R
61	MSH	MC5	MC5R
62	NPY	Y1	NPY1R
63	NPY	Y2	NPY2R
64	NPY	Y4	PPYR1
65	NPY	Y5	NPY5R
66	NPY	Y6	NPY6R
67	Neurotensin	NT1	NTSR1
68	Neurotensin	NT2	NTSR2
69	Opioid	μ	OPRM1
70	Opioid	δ	OPRD1
71	Opioid	κ	OPRK1
72	Nociceptin	ORL-1	OPRL1
73	Orexin	OX1	HCRTR1
74	Orexin	OX2	HCRTR1
75	Oxytocin	OT	OXTR
76	Somatostatin	SST1	SSTR1
77	Somatostatin	SST2	SSTR2
78	Somatostatin	SST3	SSTR3
79	Somatostatin	SST4	SSTR4
80	Somatostatin	SST5	SSTR5
81	Tachykinin	NK1	TACR1
82	Tachykinin	NK2	TACR2
83	Tachykinin	NK3	TACR3
84	TRH	TRHR	TRHR
85	VIP	VPAC1	VIPR1
86	VIP	VPAC2	VIPR2
87	Vasopressin	V1a	AVPR1A
88	Vasopressin	V1b	AVPR1B
89	Vasopressin	V2	AVPR2
90	Adenosine	A1	ADORA1
91	Adenosine	A2A	ADORA2A
92	Adenosine	A2B	ADORA2B
93	Adenosine	A3	ADORA3
94	Purine	P2Y1	P2RY1
95	Purine	P2Y2	P2RY2
96	Purine	P2Y4	P2RY4
97	Purine	P2Y6	P2RY6
98	Purine	P2Y11	P2RY11
99	Cannabinoid	CB1	CNR1
100	Cannabinoid	CB2	CNR2

Expression data were available from four or more brains in 86% of the 169 brain regions, and 53% of the regions were represented in all six brains. Only four brain regions (2%) were represented by a single brain. The median number of mRNA probes per gene was 3.

The initial data processing was performed in Mathematica 10.4 (Wolfram Research, Inc.). The mean expression values of each brain region were obtained by averaging across all probes in each of the brains, followed by averaging of the obtained means across all brains.

**Fig. 1:**
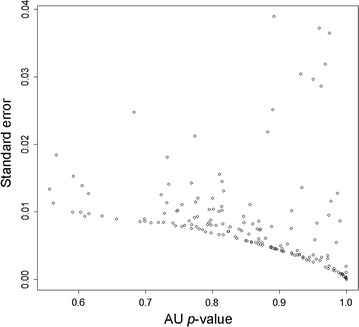
The estimated standard errors of the approximately unbiased *p* values (AU)

Each brain region was assumed to be a 100-dimensional vector (where each neurotransmitter receptor represented a dimension), and the standard Euclidean metric was used to measure the functional distance between any two regions. The hierarchical clustering analysis used the “average” agglomerative method and was performed in R 3.3.1 (The R Foundation for Statistical Computing) using the package *pvclust* [23]. This package uses multiscale bootstrap resampling [24] to estimate the approximately unbiased (AU) probability of detected clusters. It has been demonstrated that the AU is superior to the ordinary bootstrap probability (BP), and its high value (e.g., >0.95) provides strong evidence that the detected cluster exists in the population [25]. The number of bootstrap replications was 10,000, and the sample sizes ranged from 0.5 to 1.4 of the original size (with a step of 0.1). With these settings, most of the standard errors of the AU *p* values did not exceed 0.02 (Fig. 1). AU *p* values were used to guide interpretations, but no arbitrary cut-offs were used. This general approach has been successfully used in a number of applications, including GPCR-based profiling of human tissues [26], classification of tumors based on gene expression [25], and analysis of regional gene expression patterns in avian brains [27, 28].

## Results

**Fig. 2:**
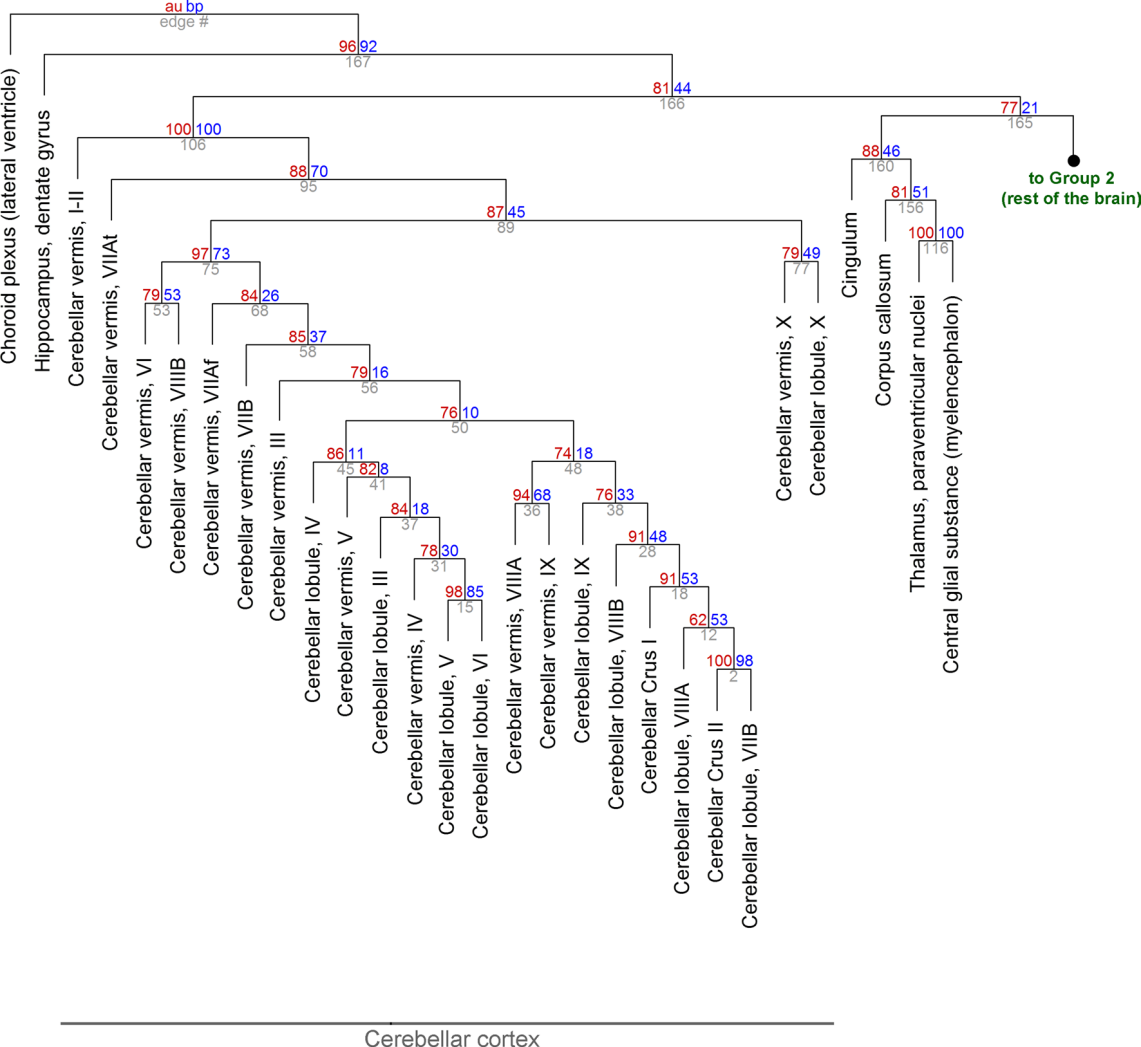
The hierarchical clustering of brain structures (Group 1). At each cluster, the *top two numbers* represent the estimated approximately unbiased *p* value (AU, *left*, *red*, %) and bootstrap probability value (BP, *right*, *blue*,  %), and the cluster identification number is indicated below (*gray*). The right branch of cluster #165 (Group 2) is expanded in Fig. 3. For compactness, the length of the branches does not represent distances. The “lobules” refer to the hemispheric parts of the cerebellar lobules

**Fig. 3:**
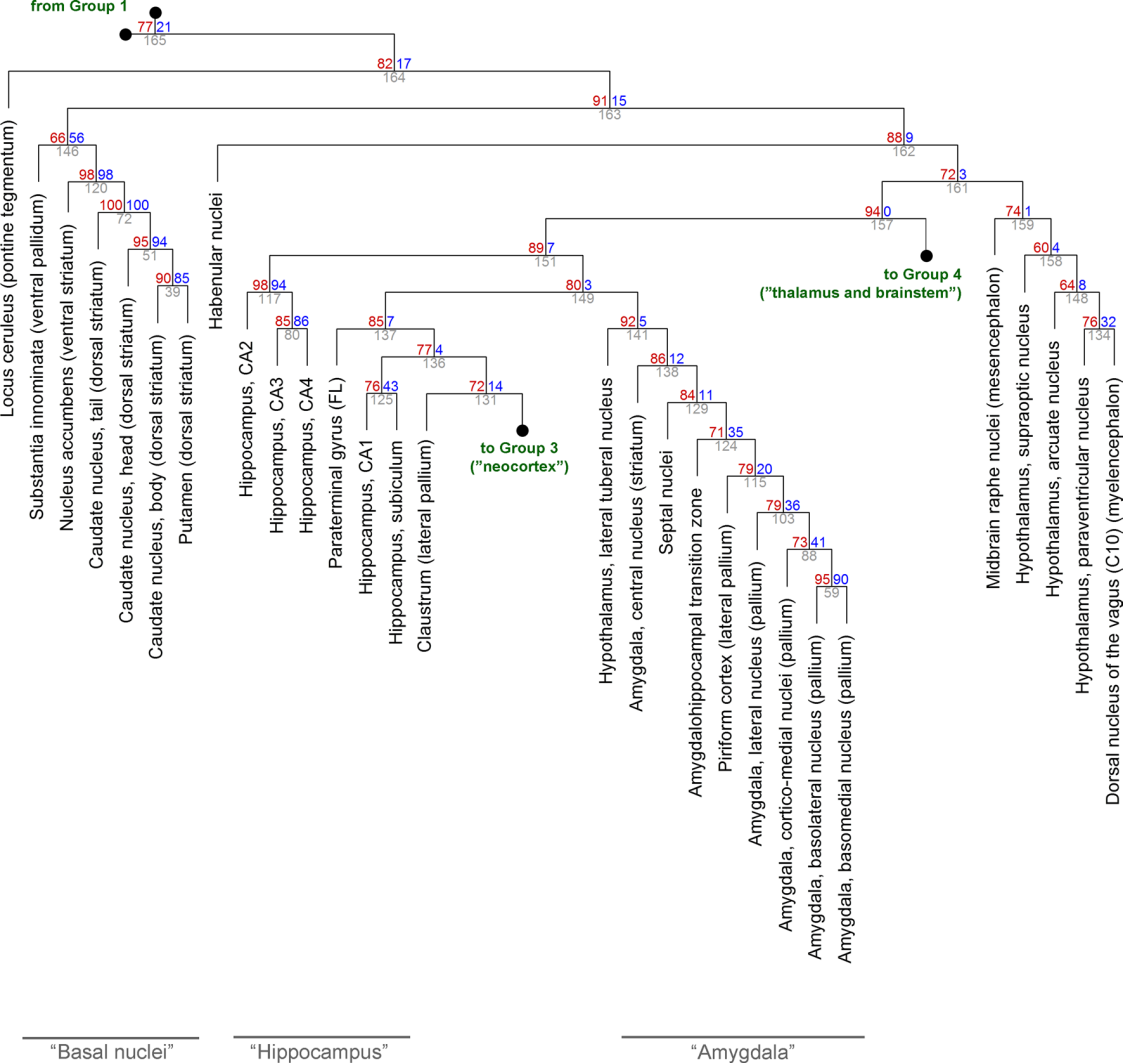
The hierarchical clustering of Group 2. At each cluster, the *top two numbers* represent the estimated approximately unbiased *p* value (AU, *left*, *red*, %) and bootstrap probability value (BP, *right*, *blue*, %), and the cluster identification number is indicated below (*gray*). The right branches of cluster #131 (Group 3) and cluster #157 (Group 4) are expanded in Figs. 4 and 5, respectively. For compactness, the length of the branches does not represent distances. The term “basal nuclei” is replacing the traditional but anatomically inaccurate term “basal ganglia” [30]

**Fig. 4:**
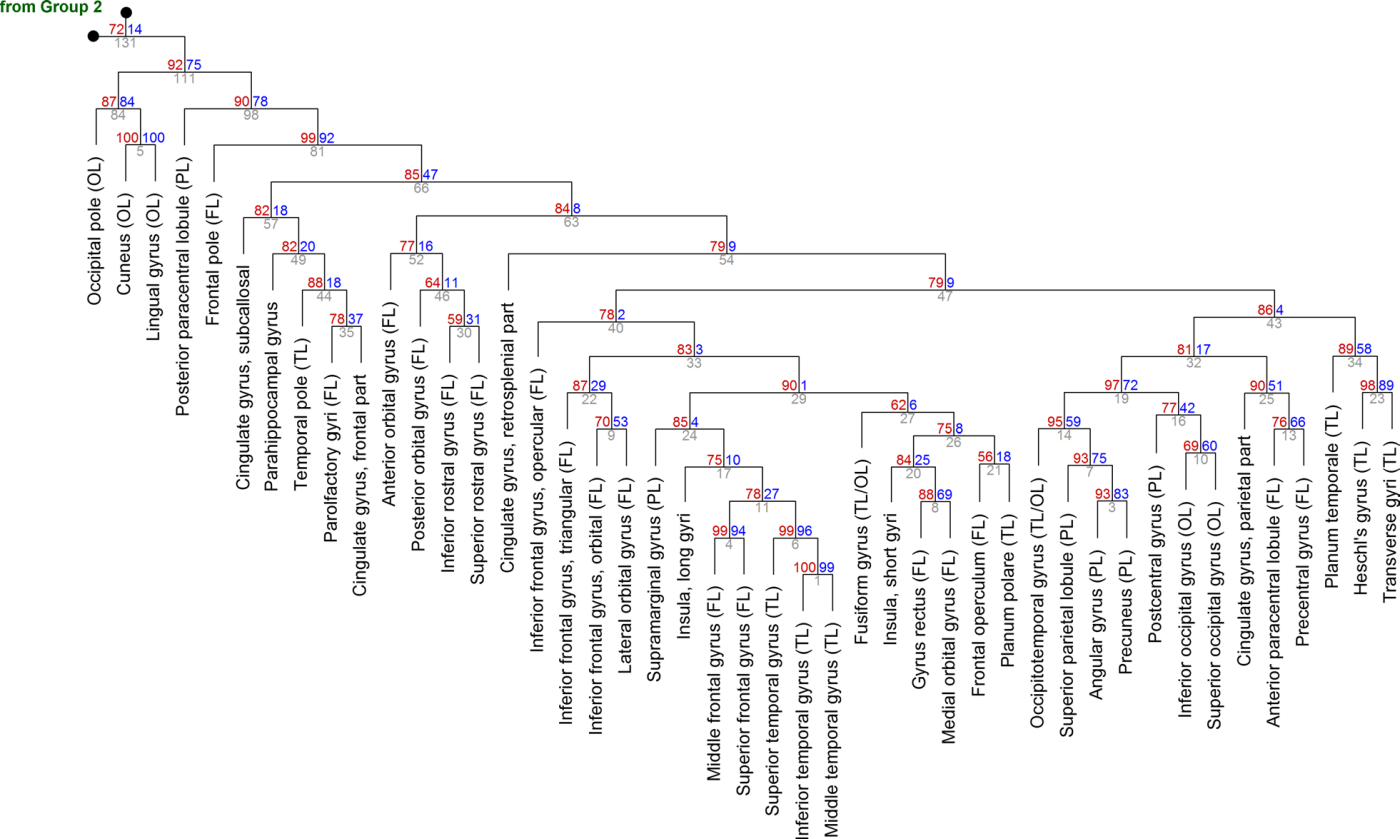
The hierarchical clustering of Group 3 (from Fig. 3). At each cluster, the *top two numbers* represent the estimated approximately unbiased *p* value (AU, *left*, *red*, %) and bootstrap probability value (BP, *right*, *blue*, %), and the cluster identification number is indicated below (*gray*). For compactness, the length of the branches does not represent distances. *FL* frontal lobe, *OL* occipital lobe, *PL* parietal lobe, *TL* temporal lobe

**Fig. 5:**
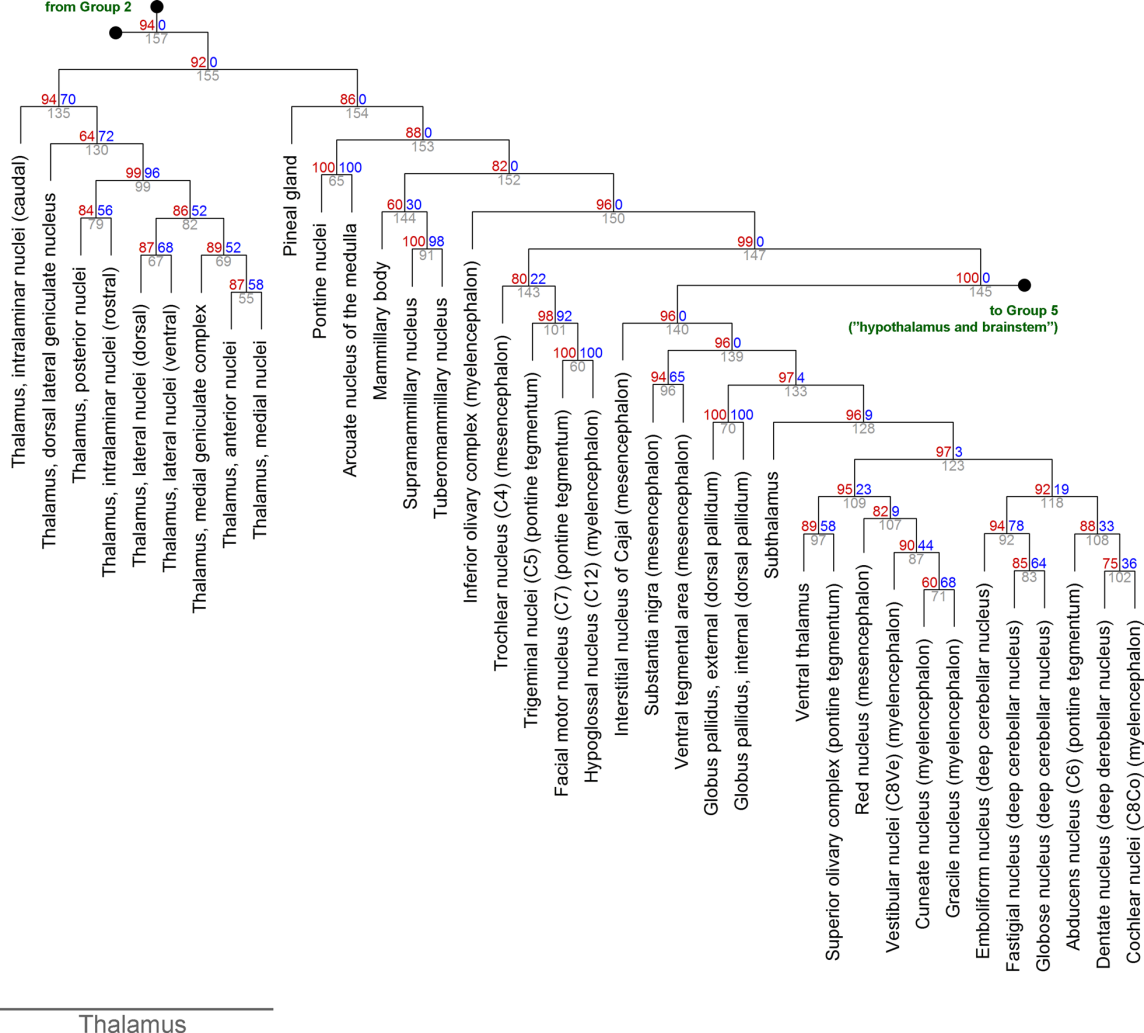
The hierarchical clustering of Group 4 (from Fig. 3). At each cluster, the *top two numbers* represent the estimated approximately unbiased *p* value (AU, *left*, *red*, %) and bootstrap probability value (BP, *right*, *blue*, %), and the cluster identification number is indicated below (*gray*). The right branch of cluster #145 (Group 5) is expanded in Fig. 6. For compactness, the length of the branches does not represent distances

**Fig. 6:**
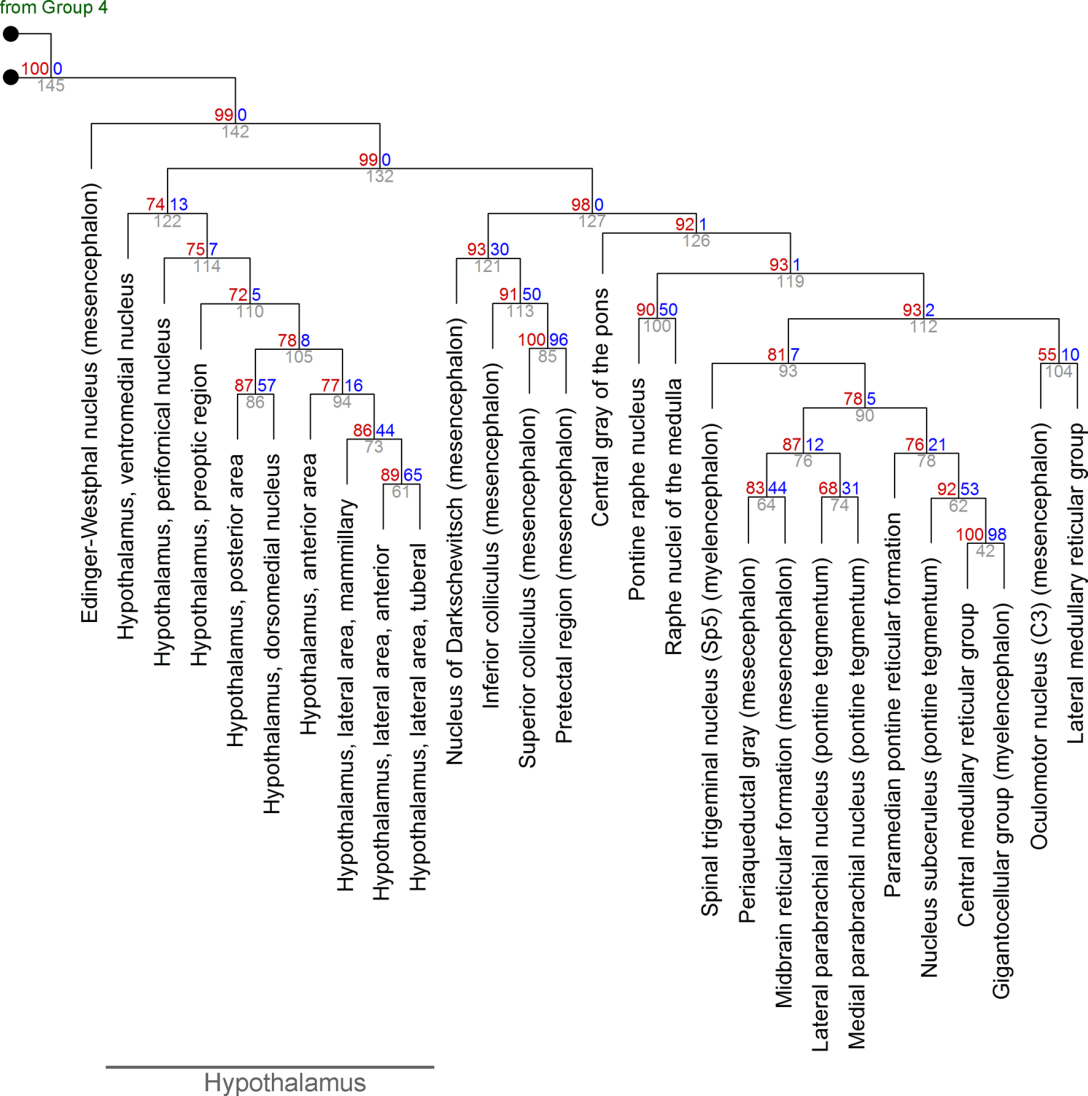
The hierarchical clustering of Group 5 (from Fig. 5). At each cluster, the *top two numbers* represent the estimated approximately unbiased *p* value (AU, *left*, *red*, %) and bootstrap probability value (BP, *right*, *blue*, %), and the cluster identification number is indicated below (*gray*). For compactness, the length of the branches does not represent distances

The results of the clustering analysis are presented in Figs. 2, 3, 4, 5 and 6. All brain regions formed a strong, highest-level cluster (#167, AU = 0.96) that excluded only one structure, the choroid plexus (Fig. 2). At the next level, this cluster split into the dentate gyrus of the hippocampus and the rest of the regions (#166, AU = 0.81). Notably, this main cluster included the pineal gland, parts of which are likely to operate outside the blood–brain barrier [29] (Fig. 5).

The main brain cluster (Fig. 2) was composed of two big clusters: the exceptionally tight cluster of the cerebellar cortex (#106, AU = 1.0) and the rest of the regions, the clustering of which was looser at this hierarchical level (#165, AU = 0.77). A large, strong sub-cluster within the cerebellar cortex (#75, AU = 0.97) excluded vermal lobules I-II (also known as the lingula) and VIIAt, as well as the entire lobule X (also known as the nodulus and the flocculus in the vermis and the hemispheres, respectively).

An unexpectedly strong functional relationship was found between the paraventricular nuclei of the thalamus and the central glial substance of the myelencephalon (#116, AU = 1.0). A somewhat looser cluster at a higher hierarchical level also included the corpus callosum and the cingulum (#160, AU = 0.88). The functional similarity among these diverse structures (representing the diencephalon, myelencephalon, and telencephalon) may be due to their physical proximity to the ventricular system. However, this cluster did not include other periventricular structures such as the periaqueductal gray, raphe nuclei, or the paraventricular nucleus of the hypothalamus.

In the remaining brain regions (Fig. 3), the locus ceruleus did not form a cluster with any other regions and appeared to be a special functional environment. This contrasted with the raphe nuclei, another “diffuse” neurotransmitter system, which were similar to many other mesencephalic and metencephalic structures, including the tectum (the superior and inferior colliculi) (Fig. 6; cluster #127, AU = 0.98). The analysis also suggested that the habenular nuclei may represent a special environment, at a slightly lower hierarchical level with respect to the locus ceruleus (Fig. 3).

The striatum formed a strong cluster that included the dorsal part (the caudate/putamen) and the ventral part (the nucleus accumbens) (Fig. 3; cluster #120, AU = 0.98). At this hierarchical level, it was not associated with the dorsal pallidum (the globus pallidus), and its association with the ventral pallidum (the substantia innominata) was weak (cluster #146, AU = 0.66).

The paleocortex (the piriform cortex), the archicortex (the hippocampus), and the neocortex formed a cluster that also included all subdivisions of the amygdala, the septal nuclei, the lateral tuberal nucleus of the hypothalamus, and the claustrum (Fig. 3; cluster #151, AU = 0.89). Among the hippocampal subdivisions, CA1 and the subiculum were most strongly associated with the neocortex (cluster #137, AU = 0.85). Notably, the central nucleus of the amygdala (of presumed striatal origin) was associated not with the striatum but with the amygdalar nuclei of presumed lateral pallial origin (the lateral nucleus, the basolateral nucleus, the basomedial nucleus, and the cortico-medial nuclei) (cluster #141, AU = 0.92).

Within the neocortical group (Fig. 4), a strong cluster was formed by cortical areas that excluded the occipital pole, the cuneus, the lingual gyrus, and the posterior paracentral lobule (cluster #81, AU = 0.99). Of the excluded regions, the cuneus and the lingual gyrus appeared to be nearly identical environments (cluster #5, AU = 1.0). Within the large neocortical cluster, the strongest sub-clusters were formed by the superior, middle, and inferior temporal gyri, with the notable exception of the transverse/Heschl gyri (cluster #6, AU = 0.99), and by the superior and middle frontal gyri (cluster #4, AU = 0.99). A functionally interesting cluster was formed by the occipitotemporal gyrus, superior parietal lobule, angular gyrus, and precuneus (cluster #14, AU = 0.95) which, extended one hierarchical step up, also included the postcentral gyrus, the inferior occipital gyrus, and the superior occipital gyrus (cluster #19, AU = 0.97).

The thalamic nuclei formed a strong cluster (Fig. 5; cluster #135, AU = 0.94), and the association of this group with the less tight hypothalamic cluster was no stronger than that with the brainstem group. Interestingly, the hypothalamic environment appeared to be strongly “mesencephalic,” not “diencephalic” (Fig. 6; cluster #142, AU = 0.99).

The four deep cerebellar nuclei were radically different from the cerebellar cortex (consistent with classic presentations [30]) and were strongly associated with some cranial nuclei (the abducence and cochlear nuclei), other related brainstem nuclei (the superior olivary complex and the red nucleus), and brainstem somatosensory nuclei (the cuneate and gracile nuclei) (Fig. 5; cluster #123, AU = 0.97). Extended three hierarchical steps up, the cluster included the subthalamus, the globus pallidus, and the substantia nigra with the ventral tegmental area (cluster #139; AU = 0.96). Another step up, it included the interstitial nucleus of Cajal (cluster #140, AU = 0.96). This mesencephalic nucleus projects directly to the oculomotor complex, but it also projects to the inferior olivary nucleus, which in turn projects to the cerebellar cortex [31].

## Discussion

Generally, the functional similarity among brain structures was consistent with classic neuroanatomical presentations and the development of the brain vesicles. The large traditionally defined groups included the telencephalon with its paleocortical, archicortical, and neocortical (isocortical) subdivisions, the thalamus and the hypothalamus, the deep cerebellar nuclei with cranial and other brainstem nuclei, the cerebellar cortex, and the brainstem. It should be emphasized that the clustering algorithm was blind to any knowledge about potential associations among the brain regions (neuroanatomical, developmental, or network-related), and that these divisions were reassembled by the algorithm based solely on the regions’ neurotransmitter receptor profiles.

The obtained clusters appeared to be strongly driven by the spatial geometry of the brain. The current view of the brain is strongly functional and emphasizes systems, networks, and long-range projections [12, 13, 32, 33]. Because the activity of some far-separated brain structures is correlated [11], their receptor profiles might too be similar. The obtained results suggest that, generally, physical proximity trumps long-range functional connectivity. This is exemplified by a large midbrain cluster (Fig. 6; cluster #127). Its structures participate in vastly different systems (e.g., vision, pain modulation, diffuse neurotransmission), but they are physically adjacent by virtue of being located in the tectum and the tegmentum. The simplicity of this result is somewhat unexpected, but it may shed new light on some unsettled problems. For example, the “amygdala” can be considered to be a highly heterogeneous complex, composed of at least a striatal (“basal nuclei-like”) part and a lateral-pallial (“piriform cortex-like”) part [31, 34, 35]. The present study cannot prove or disprove this hypothesis (which has a developmental component), but all amygdalar nuclei in the adult brain appear to be similar environments with respect to GPCR expression (Fig. 3). Also, the substantia nigra plays a key role in the function of the basal nuclei (and is sometimes considered a part of them), but it is not clear if its cellular environment is more similar to the dorsal striatum (the caudate/putamen) or the dorsal pallidum (the globus pallidus). The clustering analysis strongly suggests the latter (Fig. 5).

Care should be used in these generalizations. Adjacent regions may be difficult to dissect along strict anatomical boundaries (which can also vary from individual to individual), and some dissected brain regions may contain functionally distinct subregions that may not be captured at this level of resolution. For example, the substantia nigra is composed of the *pars compacta* and *pars reticulata*, the latter of which contains GABAergic neurons. In their morphology and connectivity, these neurons are similar to the neurons of the globus pallidus [36, 37]. Likewise, the strong separation of the Edinger–Westphal nucleus from the oculomotor nucleus (Fig. 6) may be due to the fact that the Edinger–Westphal nucleus, though associated with the oculomotor nucleus, also contains neurons that project to very different targets [38]. A recently published human brain atlas at the cellular resolution is a major improvement in the delineation of brain regions in three dimensions [39].

The close association among the precuneus, angular gyrus, superior parietal lobule, and occipitotemporal gyrus (Fig. 4) is interesting because these structures can be viewed as a subset of the default-mode network [11, 13]. However, the analysis revealed no clear functional similarity among different parts of large-scale brain networks. For example, the medial prefrontal cortex, an essential part of the default-mode network, was not clustered with the parietal structures of the network. Also, the analysis separated the anterior (frontal) cingulate cortex from the insula, even though they are thought to form a “salience” network [13]. As supported by many experimental findings, the anterior (frontal) cingulate cortex was strongly different from the posterior (parietal) cingulate cortex, the former being associated with temporal regions and the latter with occipital, parietal, and frontal regions just anterior to the central sulcus (Fig. 4).

The general functional continuity of adjacent areas does not imply that all traditional brain divisions are consistent with the obtained results. For example, the hippocampal dentate gyrus appears to be a radically different environment compared to the rest of the brain (Fig. 2). One possible explanation for this finding is that the dentate gyrus in a special region that continues to generate new neurons in adulthood [40–42]. Its unique GPCR profile may reflect mammalian brain differentiation because non-mammalian species that do not have a well-defined dentate gyrus also show hippocampal neurogenesis. It has been suggested that the dentate gyrus is a relatively “young” substructure in an “old” (archicortical) brain region [42]. Further support for this association could be obtained from other mammalian brain regions in which adult neurogenesis has been demonstrated. One such region is the ventricular-subventricular zone (V-SVZ) of the lateral ventricles that produces the rostral migratory stream to the olfactory bulb [41, 43]. Olfactory neurogenesis is well established in adult rodents; however, in the developing human brain it declines after the first two years and is virtually absent in adulthood [41, 43, 44]. A recent study has provided evidence that new neurons may be added to the adult human striatum [45], but this finding is inconsistent with another report [46] and needs further verification [43].

Functionally, the hypothalamus appears to be more associated with the midbrain than with the thalamus (Fig. 6). This does not interrupt physical continuity and requires only a rostro-caudal tilt of the dorso-ventral plane. In a different context, a similar tilt is used to explain why thalamocortical projections have to pass through the ventral thalamus, which in the developing brain is shifted rostrally. The thalamic reticular nucleus, an essential component of thalamic function, is a major derivative of the ventral thalamus [47]. Also, the midbrain and the hypothalamus are connected through the ventral branch of the ascending reticular activating system (ARAS) which controls global brain functions, including wakefulness and sleep [48].

The Allen Human Brain Atlas provides fine-grained information about the expression of a large set of genes in many brain regions. However, the current specimen set is small (six brains). The obtained results should be interpreted with caution, especially when the statistical evidence for a cluster is low. An important aspect of this study is that it uses a recently developed method to assign probabilities to all clusters; this information should be included in interpretations of the results. Some low probabilities may be due to expression data that are noisy or vary considerably across individuals [14]. However, they may also reflect meaningful differences between subject groups. In particular, hierarchical clustering can be used to study sex-specific differences in receptor expression (in larger samples). A recent DTI study, based on an exceptionally large sample, has shown considerable differences between the brains of females and males with respect to their large-scale network connectivity [49].

The used data set has important limitations, some of which are discussed in a study of large-scale GPCR associations [14]. Functional GPCRs are proteins, but the analysis was based on GPCR mRNA levels. It has been estimated that, on average, mRNA levels explain only 40% of the variability in protein levels, and that protein amounts are strongly controlled by translation [50]. However, protein amounts of a GPCR can still be misleading because its functional effects may crucially depend on its embedding in different membrane domains [51], post-translational modifications [52], internalization [53], and heteromerization with other GPCRs [54, 55]. These processes are essential for understanding the functional strength of a receptor in a given region, but they also limit the accuracy of protein detection methods. In contrast, mRNA amounts can be measured with high accuracy, and these methods can be easily automated to achieve high throughput.

It should also be noted that similar receptor levels may be present in different cell types (e.g., neurons, microglia, endothelial cells) and in different cellular domains (e.g., presynaptically or postsynaptically, on proximal or distal dendrites). Also, some GPCRs can activate several different signal transduction pathways. This suggests that similarity between two mRNA profiles can be meaningful only if interpreted in the context of neuroanatomical and neuropharmacological information. In the study, this problem was mitigated by the large receptor set and the Euclidean distance metric. In this approach, two brain regions are “close” only if their mRNA levels are comparable for most of the receptors (i.e., similarity among a small number of receptors is insufficient). Since many receptors tend to be expressed in specific cell types and cellular domains, a small Euclidean distance is unlikely to be due to a structural or spatial permutation of receptors in the two regions, leading to their radically different functional states.

## Conclusions

The study presents an unbiased, hierarchical classification of human brain regions based on their GPCR expression. At this point of mammalian brain evolution, spatial proximity of brain regions tends to override long-range connectivity. However, some brain structures represent unique environments, suggesting uneven and ongoing differentiation within the central nervous system.

## Abbreviations

AU: approximately unbiased probability
BP: bootstrap probability
CA1: *Cornu Ammonis* area 1
